# Left ventricle function and post-transcriptional events with exercise training in pigs

**DOI:** 10.1371/journal.pone.0292243

**Published:** 2024-02-02

**Authors:** Stephanie L. Samani, Shayne C. Barlow, Lisa A. Freeburg, Traci L. Jones, Marlee Poole, Mark A. Sarzynski, Michael R. Zile, Tarek Shazly, Francis G. Spinale

**Affiliations:** 1 Cell Biology and Anatomy, University of South Carolina School of Medicine, Columbia, SC, United States of America; 2 Columbia VA Health Care System, Columbia, SC, United States of America; 3 Department of Exercise Science, Arnold School of Public Health, University of South Carolina, Columbia, SC, United States of America; 4 Division of Cardiology, RHJ Department of Veterans Affairs Medical Center, Medical University of South Carolina, Charleston, SC, United States of America; 5 College of Engineering and Computing, University of South Carolina, Columbia, SC, United States of America; 6 Cardiovascular Translational Research Center, University of South Carolina, Columbia, SC, United States of America; Juntendo University: Juntendo Daigaku, JAPAN

## Abstract

**Background:**

Standardized exercise protocols have been shown to improve overall cardiovascular fitness, but direct effects on left ventricular (LV) function, particularly diastolic function and relation to post-transcriptional molecular pathways (microRNAs (miRs)) are poorly understood. This project tested the central hypothesis that adaptive LV remodeling resulting from a large animal exercise training protocol, would be directly associated with specific miRs responsible for regulating pathways relevant to LV myocardial stiffness and geometry.

**Methods and results:**

Pigs (n = 9; 25 Kg) underwent a 4 week exercise training protocol (10 degrees elevation, 2.5 mph, 10 min, 5 days/week) whereby LV chamber stiffness (K_C_) and regional myocardial stiffness (rK_m_) were measured by Doppler/speckle tracking echocardiography. Age and weight matched non-exercise pigs (n = 6) served as controls. LV K_C_ fell by approximately 50% and rK_m_ by 30% following exercise (both p < 0.05). Using an 84 miR array, 34 (40%) miRs changed with exercise, whereby 8 of the changed miRs (miR-19a, miR-22, miR-30e, miR-99a, miR-142, miR-144, miR-199a, and miR-497) were correlated to the change in K_C_ (r ≥ 0.5 p < 0.05) and mapped to matrix and calcium handling processes. Additionally, miR-22 and miR-30e decreased with exercise and mapped to a localized inflammatory process, the inflammasome (NLRP-3, whereby a 2-fold decrease in NLRP-3 mRNA occurred with exercise (p < 0.05).

**Conclusion:**

Chronic exercise reduced LV chamber and myocardial stiffness and was correlated to miRs that map to myocardial relaxation processes as well as local inflammatory pathways. These unique findings set the stage for utilization of myocardial miR profiling to identify underlying mechanisms by which exercise causes changes in LV myocardial structure and function.

## Introduction

Exercise training has been shown to reduce cardiovascular risk [1] and improve left ventricular (LV) function [2]. Specifically, exercise training can increase LV ejection fraction (EF) and reduce resting and exercise heart rate [3, 4]. These effects would imply that improved LV filling (i.e. diastolic function) as well as LV ejection performance have improved with exercise training. LV diastolic function is highly dependent upon LV chamber stiffness [5, 6], however, the direct effects of an exercise training protocol on LV stiffness properties are not well understood and may be due in part to confounding factors in clinical studies such as comorbidities, differences in the exercise protocols, and the complexity of measurements. Pigs have been successfully utilized for exercise training studies with respect to cardiovascular function [7, 8]. Accordingly, the first goal of the present study was to examine LV function, in particular LV stiffness properties, in a pig model before and following an exercise training protocol.

MicroRNAs (miRs) constitute an important post-transcriptional regulatory pathway primarily by binding to mRNA transcripts and inhibiting gene expression [9, 10]. Changes in specific miRs have been reported with exercise training and LV remodeling, but are usually focused upon a specific miR target or pathway or rely on miR profiles extracted from plasma or skeletal muscle [11–13]. However, the relationship between changes in LV function and miR profiles, particularly to that of LV stiffness properties, with exercise training is not well established. Therefore, the second goal of this study was to utilize a previously established cardiovascular focused miR array [14] and determine whether and to what degree specific shifts in myocardial miR profiles occur with exercise training in pigs. This project tested the central hypothesis that a reduction in LV stiffness properties occur with exercise training and is associated with a shift in myocardial miRs that regulate pathways and components which contribute to changes in LV stiffness.

## Methods

### Exercise protocol

Yorkshire pigs (3 months, n = 9; Palmetto Research Swine, Reeseville, South Carolina) utilized were castrated males and for the purposes of this initial study, sex dependent differences in response to exercise were not considered. Pigs acclimated to the treadmill for seven days prior to initiating the exercise protocol. Baseline LV echocardiograms were acquired within 4 hours prior to the initiation of the exercise protocol. Final LV echocardiograms were obtained 4 days after the completion of the exercise protocol. An additional group of Yorkshire pigs (male, castrated, n = 6; Palmetto Research Swine, Reeseville, South Carolina) of matched age and weight were included to obtain referent control echocardiographic images as well as LV myocardium for comparative analysis. All animals were treated and cared for in accordance with the National Institutes of Health *Guide for the Care and Use of Laboratory Animals* (*Eighth Edition*. Washington, DC: 2011), and all protocols were approved by the University of South Carolina School of Medicine and WJB Dorn VA Institutional Animal Care and Use Committee. To minimize pain and distress the following steps were taken. First, for the non-invasive echocardiographic studies, the potential for stress/anxiety was minimized through administration of oral diazepam (200mg) one hour prior to the imaging procedure and supplemental midazolam (0.5–0.6mg/kg) administered intramuscularly at the time of the echocardiogram. Second, the treadmill protocol was performed under the direct supervision of the Attending Veterinarian (SB, University of South Carolina). Thirdly, the terminal procedures were performed under a full surgical plane of anesthesia using 5% isoflurane.

### LV structure and function

All LV function and subsequent analyses was performed in a blinded analysis and the code was not broken until full study completion. LV echocardiograms were performed on exercised pigs during the study (GE Vivid E9 with XDclear Ultrasound System: M5S [1.5 to 4.6 Hz] transducer probe; GE, Boston, MA). LV dimensions and functions were assessed by 2-dimensional and M-mode echocardiographic studies [15]. From the obtained LV measurements, LV end-diastolic volume (EDV), end-systolic volume (ESV), and EF were calculated using the biplane method of disks. LV wall thickness was determined and LV mass computed using conventional formulae [16]. Pulmonary capillary wedge pressure was computed using conventional Doppler methods [17]. Aortic valve pressure gradient was determined by Doppler ultrasound [18]. Speckle tracking echocardiography (STE) was also performed on the acquired ultrasound images using established approaches [19]. LV mass and EDV were indexed to body surface area (BSA) whereby the BSA was determined using a standard approach [20].

#### LV regional myocardial and chamber stiffness

Using STE on the short-axis view, LV posterior region circumferential myocardial strain (ε) was computed as:

$$\varepsilon =\frac{L^{ED}-L^{ES}}{L^{ES}}$$ (1)

where L^ED^ and L^ES^ refer to the lengths of a circumferentially-oriented segment (within the posterior wall) length at end diastole (ED) and end systole (ES), respectively. Thus, this segmental measure considers ES as the reference configuration, and reflects a linearized strain measure to characterize the regional deformation at ED. The mean regional circumferential wall stress (σ_ϴ_) at ED was then computed as:

$$\sigma_{ϴ}=\frac{Pr}{t}$$ (2)

where P is the LV pressure, r is the LV inner radius, and t is the posterior wall thickness. We assumed LV pressure at ES was zero and at ED was equivalent to pulmonary capillary wedge pressure and computed the associated strain and stress values for both LV states. The slope of the line between these two points (corresponding to ES and ED) in the circumferential stress-strain plane, developed in posterior region, was used to compute regional myocardial stiffness (rK_m_) [21] as:

$${rK}_{m}=\frac{\sigma_{ϴ}}{\varepsilon }$$ (3)

LV chamber stiffness (K_C_) was computed as:

$$Kc=\frac{PCWP}{Volume\;Strain}$$ (4)

in which volume strain was defined as the difference between EDV and ESV divided by the ESV.

### LV sampling

After completion of the protocol, and under a full surgical plane of anesthesia (5% isoflurane), the LV was harvested and the transmyocardial sections of the posterior region placed in RNAlater (Qiagen, Valencia, CA) and stored at -80⁰C until testing was performed.

### miR extraction & profiling

LV samples (30 mg) underwent miR extraction (miRNeasy Mini cat # 217084, Qiagen, Valencia, CA) and the miR pool checked for quality (Agilent RNA 6000 Nano Kit Santa Clara, CA). The miR pool was then reverse transcribed (miScript II RT HiSpec Kit cat # 218161, Qiagen, Valencia, CA). This study utilized a custom pig cardiovascular miR array (cat # 331221, MIHS-113Z, Qiagen, Valencia, CA), which contained 84 individual miRs. The resulting cDNA from miRs was used for SYBR Green PCR (miScript SYBR Green PCR Kit cat # 218073, Qiagen, Valencia, CA). Quantitative RT-PCR was performed (Bio-Rad CFX96 Touch) according to the vendor protocol. The maximum threshold cycle (C_T_) for detection was set at 35 C_T_s. C_T_ values of the mean for SNORD42B, SNORD69, SNORD61, SNORD68, SNORD96A, and RNU6-6P were used as reference values for normalization.

### Data analysis

LV function and geometry at the beginning and completion of the exercise protocol was first examined by paired, two-tailed *t*-tests. Next, LV function and geometry was compared between age/weight matched referent controls using unpaired, two-tailed *t*-tests. In the first level of miR analysis, individual miRs with a greater than or equal to two fold-change values as a result of exercise and statistically significant miRs were identified (GeneGlobe Data Analysis, Qiagen, Valencia, CA). The differential expression of individual miRs relative to referent control was analyzed using the comparative fold-change method [22]. In addition, individual miRs were clustered with respect to predominant functional pathways structured by a literature review (S1 and S3 Tables). The association between changes in LV regional and chamber stiffness to changes in individual miRs was performed using Pearson correlation. All statistical procedures were performed using SPSS (SPSS Software, Version 27, IBM SPSS) and *P* values of < 0.05 were considered statistically significant. Unless otherwise indicated, data are expressed as means ± SEM.

## Results

### LV geometry and function

**Fig 1:**
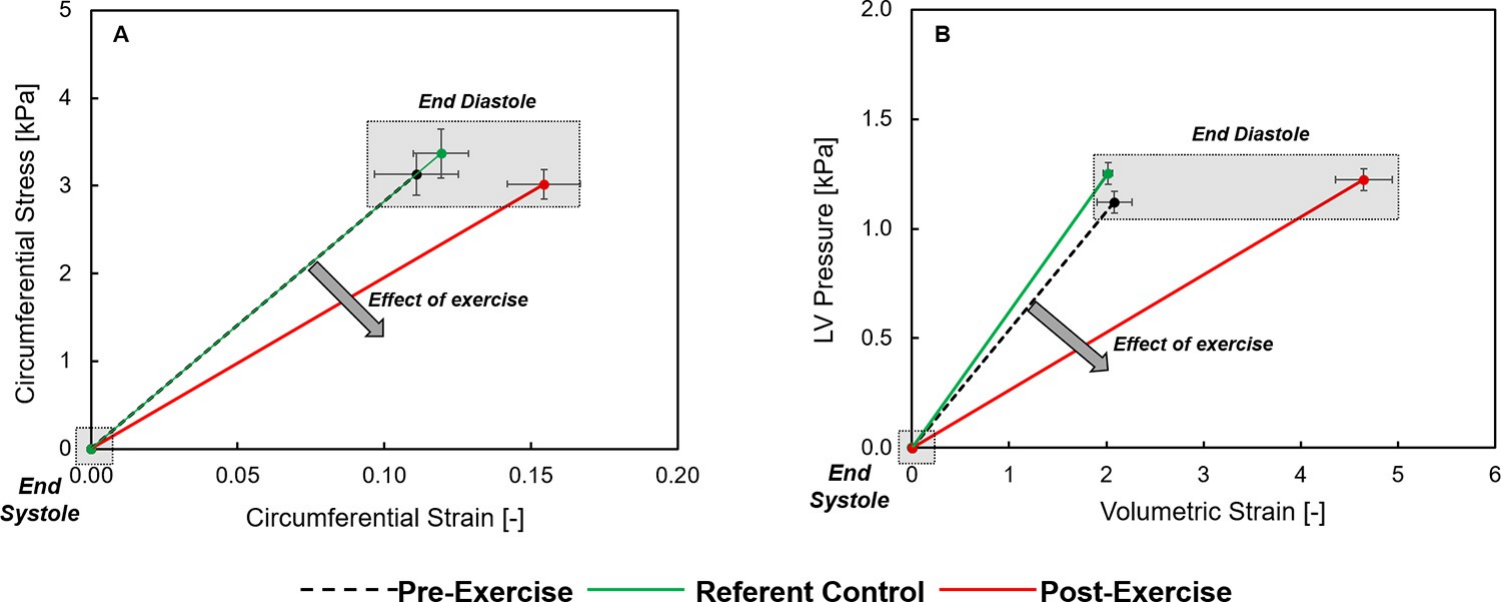
(A) LV regional circumferential stress and strain was measured by speckle tracking echocardiography (STE) in which values were calculated at the onset of diastole (end-systole) and at end diastole. A rightward and downward shift in this relation, indicative of a reduction in myocardial stiffness, occurred following the exercise protocol. (B) LV volumetric strain and pressure during diastole was computed and this relation, which is indicative of LV chamber stiffness, fell downward and to the right following the exercise protocol. Values shown are composite mean values and SEM. Summary values for LV regional myocardial stiffness and chamber stiffness are shown in Table 1.

All pigs successfully completed the exercise protocol and a summary of LV function and geometry is shown in Table 1. The values presented include pre-exercise and post-exercise values. Over the four week exercise protocol, normal weight gain occurred and accordingly, weight matched untrained referent control pigs were also included in this analysis as shown in Table 1. Resting heart rate decreased from pre-exercise values post-exercise but was similar to referent control values. The mean aortic pressure gradient, an index of aortic pressure [23], was unchanged between referent control and post-exercise values (3.15 ± 0.31 vs 4.02 ± 0.53 mmHg respectively, p = 0.273). LV mass increased following the exercise protocol, with post-exercise LV mass being higher than referent control values, and was associated with an increase in LV EDV. When indexed to BSA, the directional changes in LV mass and LV EDV remained the same. The LV posterior wall thickness (end-diastolic) to EDV ratio was similar between referent control and post exercise (0.13 ± 0.01 vs 0.12 ± 0.01 mm/mL respectively, p = 0.461). The likely reason for this index remaining within referent control values is that the LV wall thickness and LV volumes increased with the exercise protocol, consistent with concentric remodeling [24]. LV EF values were higher post-exercise from both pre-exercise and referent control values. LV posterior wall thickness increased post-exercise, as did regional ED strain. Composite plots of LV regional and chamber stiffness are shown in Fig 1, and demonstrated that both were reduced following the exercise protocol. LV rK_m_ was reduced by approximately 30% following exercise and LV K_C_ decreased by approximately 50% (Table 1).

**Table 1:** LV function and geometry: Exercise cohort.

	Pre-Exercise	Referent Control	Post-Exercise
Body Weight [Kg]	16.0 ± 0.5	25.6 ± 0.8*	24.9 ± 0.8*
HR [beats/min]	124 ± 5.2	107 ± 5.5	101 ± 2.8*
**LV**			
LV Mass [g]	69.4 ± 3.8	110.5 ± 5.5*	143.2 ± 6.5*^,^^#^
LV Mass-i	154.3 ± 8.9	179.1 ± 6.4	238.2 ± 12.3*^,^^#^
LV EDV [mL]	40.9 ± 2.8	54.8 ± 3.1*	67.4 ± 3.3*^,^^#^
LV EDV-i [mL/m^2^]	90.4 ± 5.3	89.2 ± 5.4	111.7 ± 5.1*^,^^#^
LV Mass/LV EDV	1.74 ± 0.12	2.05 ± 0.15	2.13 ± 0.06*
LV Wall Thickness/LV EDV [mm/mL]	0.16 ± 0.01	0.13 ± 0.01	0.12 ± 0.01*
LV EF [%]	66.7 ± 1.9	66.4 ± 1.8	82.0 ± 0.7*^,^^#^
LV Chamber Stiffness [kPa]	0.56 ± 0.04	0.60 ± 0.06	0.27 ± 0.02*^,^^#^
**Posterior LV Region**			
ED Wall Thickness [mm]	6.3 ± 0.3	7.2 ± 0.3	8.3 ± 0.3*^,^^#^
ED Circumferential Strain [%]	11.1 ± 1.4	11.9 ± 0.9	15.5 ± 1.3*^,^^#^
ED Wall Stress [kPa]	3.1 ± 0.2	3.4 ± 0.3	3.0 ± 0.2
Regional Myocardial Stiffness [kPa]	31.4 ± 3.8	29.1 ± 3.0	20.8 ± 2.3*^,^^#^

Sample sizes: Pre-exercise (n = 9); Post-Exercise (n = 9); Referent Control (n = 6).

HR = Heart Rate; BSA = Body Surface Area; EDV = End-Diastolic Volume; EDVi = End-Diastolic Volume Index; EF = ejection fraction; ED = End-Diastolic; i = indexed to BSA

All values reported as mean ± SEM

*p≤0.05 vs pre-exercise

^#^p≤0.05 vs referent control.

### miR profiles following exercise

**Fig 2:**
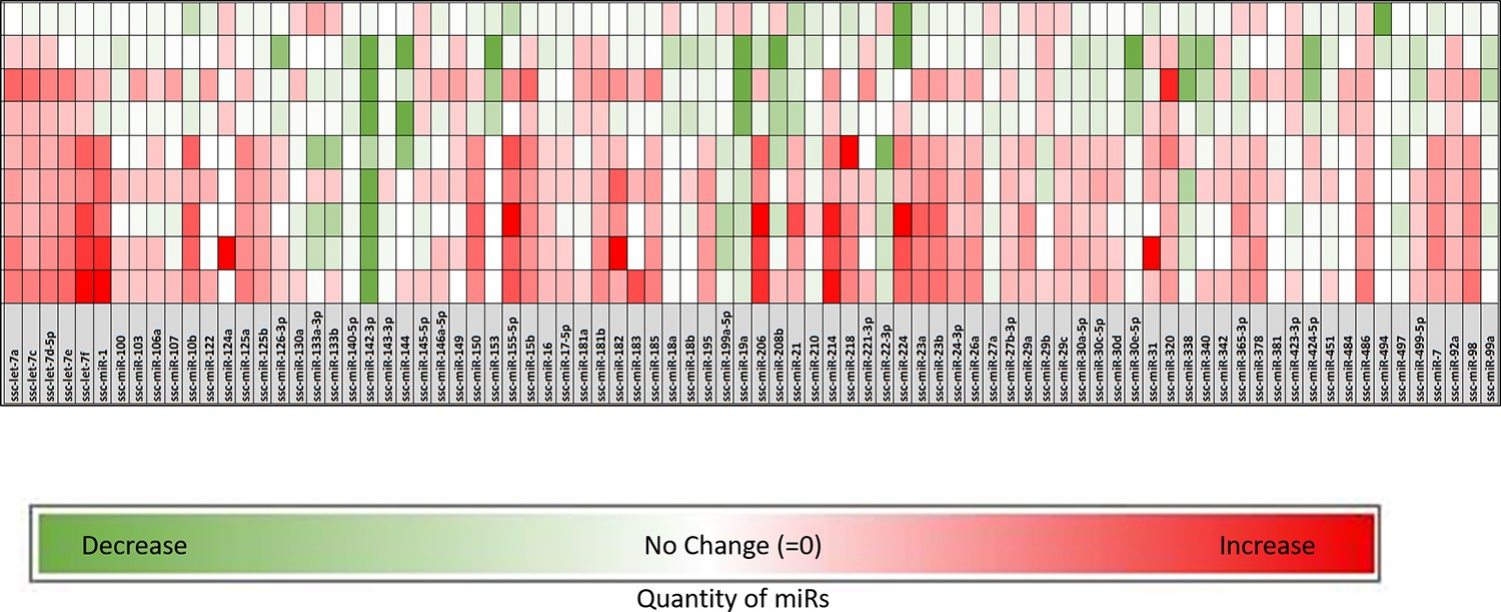
A heat map was generated for all the miRs utilized in the array, as a function of a fold change from referent control values. The rows represent individual pig values following the exercise protocol. A number of miRs changed as a function of exercise within the LV myocardium and were selected for further analysis as indicated in Table 2.

A heat map was generated as a function of referent control values for all of the included miRs (Fig 2). Several miRs changed following the exercise protocol, and those with statistically significant changes or a 2-fold or higher change were selected for further analysis (Table 2). While it must be recognized that post-transcriptional regulation by individual miRs encompasses multiple targets, predominant functional domains relevant to myocardial remodeling and function can be identified [25, 26]. This approach, and a literature review, was utilized to generate Table 2 and thus demonstrated shifts in miRs following exercise from several functional domains. A total of 25 miRs were identified as significantly (p<0.05) upregulated (56%) or downregulated (44%) following the exercise protocol. A cluster of miRs relative to myocardial growth were altered with exercise. For example, miR-199a decreased with exercise and has been identified in past studies to be altered with LV hypertrophy [27]. Although there was an even split between positive and negative fold change values within the miRs assigned to the functional domain for myocardial growth, two miRs in particular, miR-19a and miR-144, had large, significant fold decreases in expression following exercise (Table 2). Another cluster of miRs increased with exercise and have been associated with changes in LV myocardial extracellular matrix (ECM) growth and accumulation. Specifically, miR-214 and miR-155 had 3-fold increases in expression and have been previously identified as regulators of cardiac fibrosis [28, 29]. In addition, a number of miRs associated with inflammation were altered with exercise including miR-155 and miR-206, both of which were identified for their potential roles as circulatory biomarkers in inflammatory cardiomyopathy [30].

**Table 2:** Distribution of miRs with a greater than two fold-change.

miR	Fold-Change	p-value	Differentiation and Development	Disease and Dysfunction	Growth	Injury
**ssc-let-7a**	2.86 ± 0.68	0.005		x		x
**ssc-let-7c**	2.36 ± 0.66	0.022		x	x	x
**ssc-let-7d**	2.61 ± 0.66	0.008	x	x		
**ssc-let-7e**	2.68 ± 0.88	0.019		x		
**ssc-let-7f**	3.95 ± 1.37	0.008		x		x
**ssc-miR-1**	3.59 ± 1.64	0.034	x	x	x	x
**ssc-miR-15b**	2.98 ± 0.75	0.011		x		x
**ssc-miR-19a**	-7.63 ± 3.18	0.026		x	x	x
**ssc-miR-22**	-3.07 ± 0.92	0.023		x	x	x
**ssc-miR-23a**	2.02 ± 1.13	0.106			x	x
**ssc-miR-23b**	2.36 ± 0.99	0.030		x	x	
**ssc-miR-30e**	-3.28 ± 1.08	0.018			x	x
**ssc-miR-31**	6.96 ± 6.47	0.177		x	x	x
**ssc-miR-98**	2.16 ± 0.92	0.032	x	x	x	x
**ssc-miR-99a**	-2.43 ± 0.46	0.002			x	x
**ssc-miR-124a**	17.81 ± 17.18	0.175	x	x	x	x
**ssc-miR-133a**	-2.02 ± 1.00	0.079	x	x	x	x
**ssc-miR-142**	-23.39 ± 9.91	0.022	x	x		x
**ssc-miR-144**	-6.20 ± 2.90	0.031	x		x	x
**ssc-miR-153**	-9.49 ± 5.01	0.077				x
**ssc-miR-155**	3.45 ± 1.69	0.034		x	x	x
**ssc-miR-181b**	2.14 ± 0.56	0.021		x		x
**ssc-miR-182**	3.69 ± 2.29	0.110		x		x
**ssc-miR-199a**	-2.44 ± 0.67	0.020	x		x	x
**ssc-miR-206**	3.36 ± 1.80	0.054	x		x	x
**ssc-miR-208b**	-3.93 ± 1.71	0.047		x	x	x
**ssc-miR-214**	3.71 ± 1.35	0.028		x	x	x
**ssc-miR-320**	2.90 ± 0.87	0.022			x	x
**ssc-miR-338**	-3.55 ± 0.99	0.022			x	x
**ssc-miR-340**	-2.13 ± 0.96	0.038			x	x
**ssc-miR-424**	-2.37 ± 1.14	0.094	x	x	x	x
**ssc-miR-486**	2.59 ± 0.38	0.001			x	x
**ssc-miR-494**	-5.83 ± 3.53	0.063				x
**ssc-miR-497**	-2.19 ± 0.35	0.001	x		x	x

Distribution of miRs with a greater than two fold change organized by functional domain from exercised pigs (n = 9). All values reported as mean fold change ± SEM.

In order to examine the relationship between the miRs that changed with exercise (Table 2) with changes in LV regional myocardial stiffness and chamber stiffness, a correlation analysis was performed (S2 Table). A total of 8 miRs were significantly and positively correlated to either LV regional or chamber stiffness (Table 3). All 8 miRs decreased with exercise, which was associated with concomitant reductions in LV stiffness properties. The strongest correlation was observed between miR-142 and LV chamber stiffness. It is noteworthy to recognize that miR-142 is the only miR identified as having a significant association that does not belong to the myocardial growth functional group. Finally, miR-30e mapped to several metallopeptidases which would also potentially influence ECM structure and function [31]. Specifically, miR-30e likely affects post-transcriptional regulation of the metallopeptidase. Both miR-99a and miR-497 mapped to heparin sulfate proteoglycans, which have been implicated in myocardial healing and protection [32]. A literature review (S3) was utilized to place the 8 miRs that correlated to LV stiffness indices and demonstrated regulation of processes involved in adaptive remodeling such as inflammation, ECM remodeling and calcium handling (Table 4). This study mapped the specific changes in myocardial miR levels to potential functional domains, identified a relationship to LV diastolic function and, as outlined in the following paragraph, identified relevance to localized myocardial inflammation.

**Table 3:** Correlation between myocardial miR levels and stiffness indices.

miR	Regional Myocardial Stiffness		LV Chamber Stiffness	
	Correlation Coefficient	p-value	Correlation Coefficient	p-value
**ssc-let-7a**	-0.303	0.273	-0.468	0.078
**ssc-let-7c**	-0.422	0.118	-0.492	0.062
**ssc-let-7d-5p**	-0.282	0.309	-0.496	0.060
**ssc-let-7e**	-0.218	0.434	-0.462	0.083
**ssc-miR-15b**	-0.282	0.308	-0.508	0.053
**ssc-miR-19a**	0.263	0.344	0.543	0.037
**ssc-miR-22**	0.283	0.307	0.646	0.009
**ssc-miR-30e**	0.579	0.024	0.671	0.006
**ssc-miR-99a**	0.515	0.050	0.638	0.010
**ssc-miR-142**	0.388	0.153	0.814	0.000
**ssc-miR-144**	0.256	0.399	0.613	0.026
**ssc-miR-199a**	0.078	0.782	0.575	0.025
**ssc-miR-208b**	0.156	0.579	0.452	0.091
**ssc-miR-320**	-0.445	0.096	-0.441	0.100
**ssc-miR-486**	-0.287	0.300	-0.496	0.060
**ssc-miR-497**	0.391	0.149	0.726	0.002

**Table 4:** miRs with association to LV stiffness indices and functional domain.

miR	Inflammation	ECM	Calcium Handling
**ssc-miR-19a**	x	x	x
**ssc-miR-22**	x	x	x
**ssc-miR-30e**	x	x	
**ssc-miR-99a**	x		
**ssc-miR-142**	x	x	x
**ssc-miR-144**	x	x	x
**ssc-miR-199a**	x	x	x
**ssc-miR-497**		x	

### miR profiles and localized inflammation

**Fig 3:**
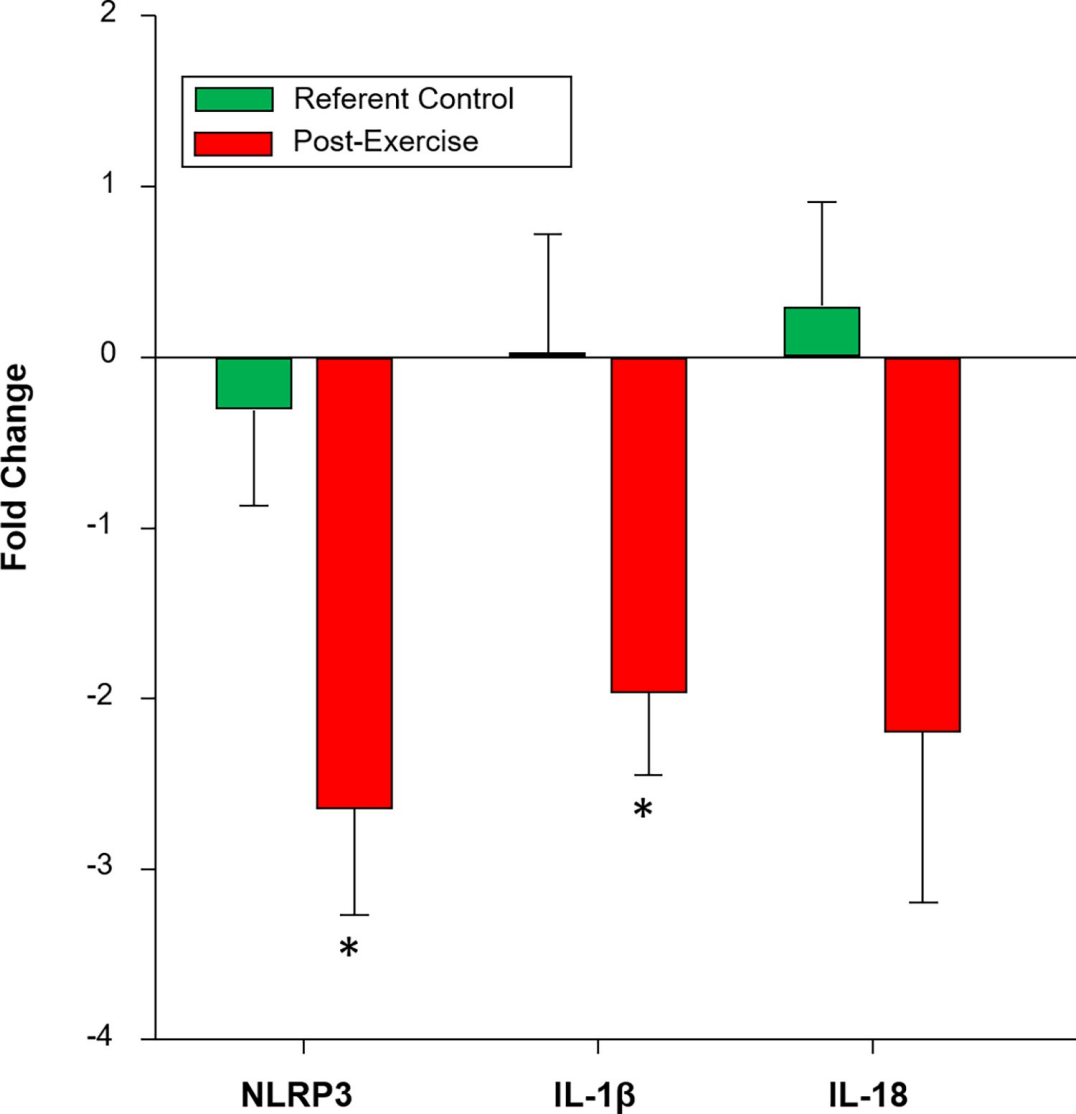
Fold change with steady state myocardial mRNA levels for components of the inflammasome. NLRP3 = NOD-LRR pyrin containing protein 3; IL-18 = interleukin 18; IL-1β = interleukin 1 beta. Values are presented as fold change mean ± SEM. *p < 0.05 vs referent control. IL-18 p = 0.083.

Several miRs mapping to local myocardial inflammatory pathways changed with exercise, which included miR-22 and miR-30e. Past studies have identified shifts in local inflammation with LV remodeling and exercise training [33–36]. Specifically, studies have identified the intracellular formation of the inflammasome, notably the NOD-LRR pyrin containing protein 3 (NLRP3), which in turn causes release of cytokine signaling molecules such as interleukin-1beta (IL1-β) and interleukin-18 (IL-18) [37, 38]. Using miR mapping algorithms (TargetScan-http://www.targetscan.org/vert_72/), several of the miRs which changed with exercise mapped to the NLRP3 inflammasome and its downstream effector proteins IL-1β and IL-18, including miR-22 and miR-30e. Myocardial mRNA analysis for these localized inflammatory components was performed using extraction approaches described previously [15]. Briefly, targeted qPCR was performed using individual primers for NLRP3 (TaQMan, Ss04953519_m1, ThermoFisher, Waltham, MA), IL-18 (TaQMan, Ss03391203_m1, ThermoFisher), and IL-1β (TaQMan, Ss03393804_m1, ThermoFisher). The maximum threshold cycle (C_T_) for detection was set at 35 C_T_s. The C_T_ value of GAPDH (TaQMan, Ss03375629_u1, ThermoFisher) was used as the reference gene value for normalization. An approximately 2-fold down regulation of mRNA levels for NLRP3, IL-1β, and IL-18 was observed following the exercise protocol (Fig 3). Furthermore, a strong positive correlation was observed between mRNA levels and LV chamber stiffness for NLRP3 (Spearman’s rho = 0.701, p<0.05), and IL-1β (Spearman’s rho = 0.643, p<0.05).

## Discussion

The overarching goal of the present study was to utilize a pig model of exercise training to examine LV function, particularly stiffness properties and the relation to changes in myocardial miRs that may be relevant to the regulation of LV structure and function. The new and unique findings from this study were three-fold. First, a standardized exercise training protocol in pigs induced LV hypertrophy as evidenced by increased LV mass, increased LV pump function as assessed by LV EF, and caused a reduction in both LV myocardial and chamber stiffness. Second, the exercise regimen caused a shift in the myocardial miR profile, in which a subset of miRs were moderately-to-strongly associated with LV stiffness properties. These miRs mapped to regulation of active relaxation (calcium handling) or to the ECM, both of which contribute to LV stiffness. Third, a subset of miRs which mapped to local inflammation were identified to change with exercise, and in particular the local inflammasome (NLRP3) pathway. The exercise protocol reduced myocardial NLRP3 expression. Taken together, these findings identified key miRs which influence LV form and function following an exercise protocol and may serve as a biomarker signature for evaluation of exercise efficacy.

### LV stiffness properties and exercise

While past studies have identified the potential benefits of a structured exercise regimen, particularly in patients with pre-existing cardiovascular conditions [39–45], the mechanistic underpinnings with respect to changes in LV structure and function remain unclear. The HF-ACTION clinical trial established that in patients with heart failure, a reduction in all cause-mortality could be achieved with exercise training [45]. In another clinical study, chronic heart failure patients underwent 2 weeks of in-hospital exercise followed by six months of home-based exercise [39]. In this study, resting HR was significantly reduced, LV mass increased, and resting LV EF and EDV were significantly improved in the exercise group- all similar findings to our present study. In a large clinical study of patients without a diagnosis of heart failure, increased physical activity was associated with reduced cardiovascular outcomes, suggestive of improved LV function [46]. However, extrapolation of the findings from the present study to a clinical context may be problematic. Specifically, the present study utilized a training protocol in young pigs in the absence of cardiovascular disease. Nevertheless, the unique findings of the present study provided a relationship betweeen a functional (LV stiffness properties) outcome and a molecular response (shifts in miRs) with an exercise protocol.

One of the underlying factors for exercise intolerance, an indication of heart failure, particularly in the absence of significant coronary artery disease, is impaired LV diastolic function due to increased LV stiffness properties [43]. However, clinical studies which have examined the effects of exercise and LV stiffness properties, both at the chamber and myocardial level, are limited. This is due to in part to problematic issues of outpatient based exercise programs and imaging modalities. In a small clinical study, patients with LV hypertrophy and evidence of increased LV filling pressures were randomized to a high-intensity exercise protocol and LV chamber stiffness was computed using LV EDV and pressure estimates [44]. At the conclusion of the year-long study LV chamber stiffness was reduced by approximately 50% in the exercise group, but was unchanged in sedentary controls. In another clinical study [47], the same group demonstrated that an exercise program could attenuate the progressive increase in LV chamber stiffness- an all too common consequence of aging and a sedentary lifestyle. While these clinical studies demonstrated a positive impact on LV chamber stiffness with a structured exercise program, the underlying contributory mechanisms for these effects remained unclear. LV chamber stiffness is determined by a summation of active and passive processes during diastole [48]. Specifically, impairment in active relaxation processes (i.e. calcium handling and myofilament interactions) will increase LV chamber stiffness. Changes in myocardial structure (such as ECM) influence passive stiffness properties, most commonly measured by LV myocardial stiffness. In the present large animal study a chronic exercise protocol reduced LV chamber stiffness as well as myocardial stiffness. These results imply that a contributory mechanism for the reduction in LV chamber stiffness observed in past clinical reports in an exercise protocol is due, at least in part, to a reduction in LV myocardial stiffness. The present study also established that a post-transcriptional mechanism for this reduction in myocardial stiffness was a reduction in specific miRs which regulate ECM and inflammation.

Several previous studies used large animal models of exercise, but were primarily focused upon mechanisms contributing to LV pump function and contractility [7, 8, 49, 50]. In one study, progressive treadmill training in pigs resulted in increased myofilament force production and computed myocardial power [7]. In another study, exercise training in pigs was shown to shift calcium myofilament sensitivity and also change active relaxation kinetics [51]. The relevance of these past studies to the present study are three-fold. First, these past studies identified increased myocyte/myofilament contractility and thus a likely contributing factor for the increased LV pump function observed in the present study following a treadmill exercise protocol. Second, these past studies identified shifts in calcium myofilament sensitivity and improved active relaxation and are likely contributing factors for the reduction in LV myocardial stiffness observed in the present study following the exercise protocol. Thirdly, as detailed in the subsequent section, the present study identified shifts in miR profiles that map to key factors in calcium handling and sensitivity and represent a potential molecular mechanism contributing to improved myocyte and myofilament function reported in past studies following an exercise protocol.

### LV myocardial miRs and exercise

Previous human studies evaluating miR levels following exercise training primarily include miR levels taken from peripheral blood samples of elite athletes with years of training experience [12, 52] or populations with pre-existing conditions such as heart failure or cancer [14, 53, 54]. There are limited studies of healthy populations that have quantified miR levels following exercise training focused upon a pre-selected, restricted number of miRs taken from peripheral blood samples [11, 55–57]. In a study of sedentary but otherwise healthy adults (HERITAGE Family Study) [58], we examined plasma miR profiles before and after 20 weeks of a cycle ergometer based exercise training protocol. There were discordant findings between our past study and the present study with respect to certain miRs. Specifically, in the 20 week exercise protocol, no changes in let-7c, miR-181b, miR-23b, miR-320, and miR-98 were observed, whereas the present study identified increased LV myocardial levels of these miRs. In another study, plasma levels of miR-1 and miR-486 were increased in endurance athletes compared to healthy controls [59]. These observations are consistent with the findings from the present study in which increased myocardial levels of miR-1 and miR-486 were observed following a treadmill-based exercise training protocol in pigs. However, as stated previously, direct comparisons between this large animal exercise study to that of past exercise studies in humans may be problematic.

To our knowledge this is the first study to quantify myocardial miR profiles in a pig model of treadmill-based exercise. Previously, pigs have been utilized to quantify miR profiles in other organs [60, 61] and as a large animal model of exercise [62], but studies implementing both miR profiling and exercise are lacking. Studies utilizing other large animal models such as horses or dogs to quantify miR profiles following exercise included either training for the purposes of weight loss in overweight animals [63] or single bouts of exercise [64]. However, several studies have utilized rodents to examine changes in miRs with respect to a defined exercise training protocol [65–68]. For example, an 8 week treadmill protocol caused an increase in myocardial levels of miR-1 and miR-206 in mice [67]. Using a swimming training protocol in rats, decreased myocardial levels of miR-208b and miR-133a were reported [65, 68]. The directional changes in myocardial miRs reported in these past studies are similar to those observed in the present study in which a large, cardiac-focused miR array was utilized.

The present study identified a cluster of 8 miRs which were correlated with LV stiffness properties, and while this does not imply a cause-effect, these miRs did map to relevant pathways which could affect LV stiffness (Table 4). For example, miR-144 and miR-142 exhibit post-transcriptional control of calcium/calmodulin serine protein kinases, which in turn would affect calcium handling within the cardiomyocyte [69]. This in turn would affect LV active relaxation, an important component which contributes to overall LV chamber stiffness. In addition, miR-144 mapped to a critical pathway which affects ECM structure and composition: TGF-β [70]. Changes in TGF- β signaling, particularly through the SMAD signaling cascade, can directly alter collagen synthesis. Specifically, SMAD-7 has been shown to exert an inhibitory effect on the TGF intracellular signaling cascade [71, 72]. Since miR-144 levels decreased with the exercise training protocol, then this may have altered TGF- β activation, and thus in turn reduced LV myocardial collagen accumulation, a key component of LV myocardial stiffness.

### LV myocardial inflammation and exercise

A localized inflammatory cascade, defined as the inflammasome, has received recent attention in the context of LV myocardial structure and function [33, 34]. Specifically, the NLRP3 inflammasome has been shown to be activated in the context of ischemia and related LV dysfunction [73]. In the present study, several miRs (miR-22, miR-30e, miR-140, miR-199a, miR-210 and miR-497) which either map to or correlate with the NLRP3 inflammasome were altered within the LV myocardium following the treadmill exercise protocol. In fact, a strong correlation was observed between NLRP3 and the downstream effector, IL-1β, with LV stiffness. In a past study miR-146a was shown to be mechanistically linked to NLRP3 in the context of spinal cord injury [74]. In hepatic fibrosis miR-21 negatively regulated a key transcription factor necessary for NLRP3 expression [75]. Finally, in dendritic cells, miR-155 has been identified to negatively regulate components of the inflammasome cascade [76]. Taken together, results from previous studies and the current investigation suggest that a treadmill exercise protocol directly affects key myocardial miRs which in turn regulate the myocardial inflammasome. However, it must be recognized that the post-transcriptional regulation of the inflammasome is complex and different miRs may interfere with this process at multiple intersections.

In the present study, a specific pattern of miRs associated with localized inflammation such as the inflammasome, were altered with exercise training in pigs. Exercise-induced inhibition of the activation of the NLRP3 inflammasome and downstream effectors IL-1β and IL-18 has been reported in mice [77, 78]. In a limited number of clinical studies, a reduction in indices of inflammasome activation have been reported following a chronic exercise protocol [79, 80]. However, the underlying mechanisms by which a reduction in the inflammasome, such as NLRP3, occurs as a function of exercise is poorly understood. The present study identified that specific miRs, such as miR-30e, may be important in the post-transcriptional regulation of the inflammasome. Moreover, the present study suggests that profiling a specific cassette of miRs may provide a basis for identifying a beneficial effect of a prescribed exercise training program on localized inflammation. Past studies have examined the effects of exercise training on plasma miR profiles and our group has associated these changes with myocardial structure-function [11–14, 30, 52–59, 81]. However, the present study quantified myocardial miR levels following a specific exercise protocol and whether and to what degree these observations can be translated to plasma, and more importantly to patients following an exercise training protocol, will require future study.

## Study limitations and summary

While the present study utilized a large animal model to examine LV function, particularly LV stiffness properties, and the relationship to myocardial miR profiles, there are several study limitations which must be recognized. First, the utilized training protocol, while standardized, did not perform oxygen saturation and consumption measurements, and therefore the relative periods of aerobic and anaerobic exercise was not determined. Second, the exercise protocol was performed in normal pigs and whether and to what degree this protocol can be utilized in the context of ischemia and pressure overload cardiac pathologies remains to be determined. Third, the pigs utilized in this study were 3 months of age and would be considered young since the age for sexual maturity is 5 months of age [82]. In addition, the pigs utilized in this study were castrated, which removes sex as a covariate in these studies. Nevertheless, this large animal model of chronic exercise did allow for assessing relevant measures of LV function, such as LV stiffness properties, which can be problematic in rodent models. Fourth, the present study quantified myocardial miRs using a large array using pre-specified normalization algorithms in terms of the total miR pool and referent normal values. This makes direct comparisons to past studies which utilized plasma samples problematic. Nevertheless, this is the first study to demonstrate a direct relationship between LV stiffness properties (both at the chamber and myocardial level) to shifts in the abundance of myocardial miRs, as well as to localized myocardial inflammation following a standardized exercise regimen. These findings may set the stage for translational studies which utilize a specific miR signature to identify exercise efficacy.

## Supporting information

S1 Table Distribution of miRs with a greater than two fold-change. (DOCX)

S2 Table Association between myocardial miR levels & stiffness indices for all miRs with a greater than 2 fold change. (DOCX)

S3 Table miRs with association to LV stiffness indices and functional domain. (DOCX)

S1 Graphical abstract (TIF)
