# Supplementary material for: Left ventricle function and post-transcriptional events with exercise training in pigs
Source: PLoS One. 2024 Feb 2;19(2):e0292243. doi: 10.1371/journal.pone.0292243 (PMC10836705; doi:10.1371/journal.pone.0292243)
Supplement: S1 Table — (DOCX) [file pone.0292243.s001.docx]

| **Supplemental Table 1: Distribution of miRs with ≥2 Fold Change According to Functionality** | | | | | | |
| --- | --- | --- | --- | --- | --- | --- |
| **miR** | **Fold-Change** | **p-value** | **Differentiation and Development** | **Disease and Dysfunction** | **Growth** | **Injury** |
| **ssc-let-7a** | 2.86 ± 0.68 | 0.005 |  | 1 |  | 2 |
| **ssc-let-7c** | 2.36 ± 0.66 | 0.022 |  | 3 | 4 | 5 |
| **ssc-let-7d** | 2.61 ± 0.66 | 0.008 | 6 | 7 |  |  |
| **ssc-let-7e** | 2.68 ± 0.88 | 0.019 |  | 8 |  |  |
| **ssc-let-7f** | 3.95 ± 1.37 | 0.008 |  | 9 |  | 10 |
| **ssc-miR-1** | 3.59 ± 1.64 | 0.034 | 11 | 12 | 13 | 14 |
| **ssc-miR-15b** | 2.98 ± 0.75 | 0.011 |  | 15 |  | 16 |
| **ssc-miR-19a** | -7.63 ± 3.18 | 0.026 |  | 17 | 18 | 19 |
| **ssc-miR-22^#^** | -3.07 ± 0.92 | 0.023 |  | 20 | 21 | 22 |
| **ssc-miR-23a** | 2.02 ± 1.13 | 0.106 |  |  | 23 | 24 |
| **ssc-miR-23b** | 2.36 ± 0.99 | 0.030 |  | 25 | 26 |  |
| **ssc-miR-30e^#^** | -3.28 ± 1.08 | 0.018 |  |  | 27 | 28 |
| **ssc-miR-31** | 6.96 ± 6.47 | 0.177 |  | 29 | 30 | 31 |
| **ssc-miR-98** | 2.16 ± 0.92 | 0.032 | 32 | 33 | 34 | 35 |
| **ssc-miR-99a** | -2.43 ± 0.46 | 0.002 |  |  | 36 | 37 |
| **ssc-miR-124a** | 17.81 ± 17.18 | 0.175 | 38 | 39 | 40 | 41 |
| **ssc-miR-133a** | -2.02 ± 1.00 | 0.079 | 42 | 43 | 44 | 45 |
| **ssc-miR-142** | -23.39 ± 9.91 | 0.022 | 46 | 47 |  | 48 |
| **ssc-miR-144** | -6.20 ± 2.90 | 0.031 | 49 |  | 50 | 51 |
| **ssc-miR-153** | -9.49 ± 5.01 | 0.077 |  |  |  | 52 |
| **ssc-miR-155** | 3.45 ± 1.69 | 0.034 |  | 53 | 54 | 55 |
| **ssc-miR-181** | 2.14 ± 0.56 | 0.021 |  | 56 |  | 57 |
| **ssc-miR-182** | 3.69 ± 2.29 | 0.110 |  | 58 |  | 59 |
| **ssc-miR-199a** | -2.44 ± 0.67 | 0.020 | 60 |  | 61 | 62 |
| **ssc-miR-206** | 3.36 ± 1.80 | 0.054 | 63 |  | 64 | 65 |
| **ssc-miR-208b** | -3.93 ± 1.71 | 0.047 |  | 66 | 67 | 68 |
| **ssc-miR-214** | 3.71 ± 1.35 | 0.028 |  | 69 | 70 | 71 |
| **ssc-miR-320** | 2.90 ± 0.87 | 0.022 |  |  | 72 | 73 |
| **ssc-miR-338** | -3.55 ± 0.99 | 0.022 |  |  | 74 | 75 |
| **ssc-miR-340** | -2.13 ± 0.96 | 0.038 |  |  | 76 | 77 |
| **ssc-miR-424** | -2.37 ± 1.14 | 0.094 | 78 | 79 | 80 | 81 |
| **ssc-miR-486** | 2.59 ± 0.38 | 0.001 |  |  | 82 | 83 |
| **ssc-miR-494** | -5.83 ± 3.53 | 0.063 |  |  |  | 84 |
| **ssc-miR-497** | -2.19 ± 0.35 | 0.001 | 85 |  | 86 | 87 |

1. Zhou X, Sun F, Luo S, et al. Let-7a Is an Antihypertrophic Regulator in the Heart via Targeting Calmodulin. *Int J Biol Sci*. 2017;13(1):22-31. Published 2017 Jan 1.
2. Chen CY, Choong OK, Liu LW, et al. MicroRNA let-7-TGFBR3 signalling regulates cardiomyocyte apoptosis after infarction. *EBioMedicine*. 2019;46:236-247.
3. Yang VK, Tai AK, Huh TP, et al. Dysregulation of valvular interstitial cell let-7c, miR-17, miR-20a, and miR-30d in naturally occurring canine myxomatous mitral valve disease. *PLoS One*. 2018;13(1):e0188617. Published 2018 Jan 9.
4. Wang Y, Chen J, Song W, et al. The Human Myotrophin Variant Attenuates MicroRNA-Let-7 Binding Ability but Not Risk of Left Ventricular Hypertrophy in Human Essential Hypertension [published correction appears in PLoS One. 2016;11(1):e0146735. Ye, Zhidong [added]]. *PLoS One*. 2015;10(8):e0135526. Published 2015 Aug 14.
5. Tolonen AM, Magga J, Szabó Z, et al. Inhibition of Let-7 microRNA attenuates myocardial remodeling and improves cardiac function postinfarction in mice. *Pharmacol Res Perspect*. 2014;2(4):e00056.
6. Wong SS, Ritner C, Ramachandran S, et al. miR-125b promotes early germ layer specification through Lin28/let-7d and preferential differentiation of mesoderm in human embryonic stem cells. *PLoS One*. 2012;7(4):e36121.
7. Wong LL, Saw EL, Lim JY, Zhou Y, Richards AM, Wang P. MicroRNA Let-7d-3p Contributes to Cardiac Protection via Targeting HMGA2. *Int J Mol Sci*. 2019;20(7):1522. Published 2019 Mar 27.
8. Li X, Wang B, Cui H, et al. let-7e replacement yields potent anti-arrhythmic efficacy via targeting beta 1-adrenergic receptor in rat heart. *J Cell Mol Med*. 2014;18(7):1334-1343.
9. Liu Y, Duan C, Liu W, et al. Upregulation of let-7f-2-3p by long noncoding RNA NEAT1 inhibits XPO1-mediated HAX-1 nuclear export in both in vitro and in vivo rodent models of doxorubicin-induced cardiotoxicity. Arch Toxicol. 2019;93(11):3261-3276.
10. Chen CY, Choong OK, Liu LW, et al. MicroRNA let-7-TGFBR3 signalling regulates cardiomyocyte apoptosis after infarction. EBioMedicine. 2019;46:236-247.
11. Huang F, Tang L, Fang ZF, Hu XQ, Pan JY, Zhou SH. miR-1-mediated induction of cardiogenesis in mesenchymal stem cells via downregulation of Hes-1 [published correction appears in Biomed Res Int. 2016;2016:8510747]. Biomed Res Int. 2013;2013:216286.
12. Karakikes I, Chaanine AH, Kang S, et al. Therapeutic cardiac-targeted delivery of miR-1 reverses pressure overload-induced cardiac hypertrophy and attenuates pathological remodeling. J Am Heart Assoc. 2013;2(2):e000078. Published 2013 Apr 23.
13. Seok H, Lee H, Lee S, et al. Position-specific oxidation of miR-1 encodes cardiac hypertrophy. Nature. 2020;584(7820):279-285.
14. Ma Q, Ma Y, Wang X, et al. Circulating miR-1 as a potential predictor of left ventricular remodeling following acute ST-segment myocardial infarction using cardiac magnetic resonance. Quant Imaging Med Surg. 2020;10(7):1490-1503.
15. Zhu Y, Yang T, Duan J, Mu N, Zhang T. MALAT1/miR-15b-5p/MAPK1 mediates endothelial progenitor cells autophagy and affects coronary atherosclerotic heart disease via mTOR signaling pathway. Aging (Albany NY). 2019;11(4):1089-1109.
16. Niu S, Xu L, Yuan Y, et al. Effect of down-regulated miR-15b-5p expression on arrhythmia and myocardial apoptosis after myocardial ischemia reperfusion injury in mice. Biochem Biophys Res Commun. 2020;530(1):54-59.
17. Miao Y, Chen H, Li M. MiR-19a overexpression contributes to heart failure through targeting ADRB1. Int J Clin Exp Med. 2015;8(1):642-649. Published 2015 Jan 15.
18. Liu K, Hao Q, Wei J, Li GH, Wu Y, Zhao YF. MicroRNA-19a/b-3p protect the heart from hypertension-induced pathological cardiac hypertrophy through PDE5A. J Hypertens. 2018;36(9):1847-1857.
19. Gao F, Kataoka M, Liu N, et al. Therapeutic role of miR-19a/19b in cardiac regeneration and protection from myocardial infarction. Nat Commun. 2019;10(1):1802. Published 2019 Apr 17.
20. Wang R, Xu Y, Niu X, et al. MiR-22 Inhibition Alleviates Cardiac Dysfunction in Doxorubicin-Induced Cardiomyopathy by Targeting the sirt1/PGC-1α Pathway. Front Physiol. 2021;12:646903. Published 2021 Apr 1.
21. Huang ZP, Chen J, Seok HY, et al. MicroRNA-22 regulates cardiac hypertrophy and remodeling in response to stress. Circ Res. 2013;112(9):1234-1243.
22. van Boven N, Akkerhuis KM, Anroedh SS, et al. Serially measured circulating miR-22-3p is a biomarker for adverse clinical outcome in patients with chronic heart failure: The Bio-SHiFT study. Int J Cardiol. 2017;235:124-132.
23. Wang K, Lin ZQ, Long B, Li JH, Zhou J, Li PF. Cardiac hypertrophy is positively regulated by MicroRNA miR-23a. J Biol Chem. 2012;287(1):589-599.
24. Bukauskas T, Mickus R, Cereskevicius D, Macas A. Value of Serum miR-23a, miR-30d, and miR-146a Biomarkers in ST-Elevation Myocardial Infarction. Med Sci Monit. 2019;25:3925-3932. Published 2019 May 27.
25. He W, Che H, Jin C, Ge S. Effects of miR-23b on hypoxia-induced cardiomyocytes apoptosis. Biomed Pharmacother. 2017;96:812-817.
26. Boureima Oumarou D, Ji H, Xu J, et al. Involvement of microRNA-23b-5p in the promotion of cardiac hypertrophy and dysfunction via the HMGB2 signaling pathway. Biomed Pharmacother. 2019;116:108977.
27. Xu H, Xu Y, Yang J, Juang J. Circulating MicroRNA-30e Predicts Left Ventricular Hypertrophy In Essential Hypertensive Patients [published online ahead of print, 2020 Dec 1]. Rev Invest Clin. 2020;10.24875/RIC.20000447.
28. Chen Y, Yin Y, Jiang H. miR-30e-5p Alleviates Inflammation and Cardiac Dysfunction After Myocardial Infarction Through Targeting PTEN. Inflammation. 2021;44(2):769-779.
29. Huang R, Chen X, Long Y, Chen R. MiR-31 promotes Th22 differentiation through targeting Bach2 in coronary heart disease. Biosci Rep. 2019;39(9):BSR20190986. Published 2019 Sep 20.
30. Ren J, Liu W, Li GC, et al. Atorvastatin Attenuates Myocardial Hypertrophy Induced by Chronic Intermittent Hypoxia In Vitro Partly through miR-31/PKCε Pathway. Curr Med Sci. 2018;38(3):405-412.
31. Wang Y, Men M, Yang W, Zheng H, Xue S. MiR-31 Downregulation Protects Against Cardiac Ischemia/Reperfusion Injury by Targeting Protein Kinase C Epsilon (PKCε) Directly. Cell Physiol Biochem. 2015;36(1):179-190.
32. Sun HH, Sun PF, Liu WY. MiR-98-5p regulates myocardial differentiation of mesenchymal stem cells by targeting TBX5. Eur Rev Med Pharmacol Sci. 2018;22(22):7841-7848.
33. Zhang BY, Zhao Z, Jin Z. Expression of miR-98 in myocarditis and its influence on transcription of the FAS/FASL gene pair. Genet Mol Res. 2016;15(2):10.4238/gmr.15027627. Published 2016 Jun 3.
34. Yang Y, Ago T, Zhai P, Abdellatif M, Sadoshima J. Thioredoxin 1 negatively regulates angiotensin II-induced cardiac hypertrophy through upregulation of miR-98/let-7. Circ Res. 2011;108(3):305-313.
35. Zhai CL, Tang GM, Qian G, et al. MicroRNA-98 attenuates cardiac ischemia-reperfusion injury through inhibiting DAPK1 expression. IUBMB Life. 2019;71(2):166-176.
36. Li Q, Xie J, Wang B, et al. Overexpression of microRNA-99a Attenuates Cardiac Hypertrophy. PLoS One. 2016;11(2):e0148480. Published 2016 Feb 25.
37. Yang SY, Wang YQ, Gao HM, Wang B, He Q. The clinical value of circulating miR-99a in plasma of patients with acute myocardial infarction. Eur Rev Med Pharmacol Sci. 2016;20(24):5193-5197.
38. Cai B, Li J, Wang J, et al. microRNA-124 regulates cardiomyocyte differentiation of bone marrow-derived mesenchymal stem cells via targeting STAT3 signaling. Stem Cells. 2012;30(8):1746-1755.
39. Zhao Y, Yan M, Chen C, et al. MiR-124 aggravates failing hearts by suppressing CD151-facilitated angiogenesis in heart. Oncotarget. 2018;9(18):14382-14396. Published 2018 Jan 12.
40. Bao Q, Chen L, Li J, et al. Role of microRNA-124 in cardiomyocyte hypertrophy induced by angiotensin II. Cell Mol Biol (Noisy-le-grand). 2017;63(4):23-27. Published 2017 Apr 29.
41. Cheng XJ, Li L, Xin BQ. MiR-124 Regulates the Inflammation and Apoptosis in Myocardial Infarction Rats by Targeting STAT3. Cardiovasc Toxicol. 2021;21(9):710-720.
42. Liu N, Bezprozvannaya S, Williams AH, et al. microRNA-133a regulates cardiomyocyte proliferation and suppresses smooth muscle gene expression in the heart. Genes Dev. 2008;22(23):3242-3254.
43. Nandi SS, Shahshahan HR, Shang Q, Kutty S, Boska M, Mishra PK. MiR-133a Mimic Alleviates T1DM-Induced Systolic Dysfunction in Akita: An MRI-Based Study. Front Physiol. 2018;9:1275. Published 2018 Oct 10.
44. Wen P, Song D, Ye H, et al. Circulating MiR-133a as a biomarker predicts cardiac hypertrophy in chronic hemodialysis patients. PLoS One. 2014;9(10):e103079. Published 2014 Oct 14.
45. Xiao Y, Zhao J, Tuazon JP, Borlongan CV, Yu G. MicroRNA-133a and Myocardial Infarction. Cell Transplant. 2019;28(7):831-838.
46. Chen ZY, Chen F, Cao N, Zhou ZW, Yang HT. miR-142-3p Contributes to Early Cardiac Fate Decision of Embryonic Stem Cells. Stem Cells Int. 2017;2017:1769298
47. Sukma Dewi I, Hollander Z, Lam KK, et al. Association of Serum MiR-142-3p and MiR-101-3p Levels with Acute Cellular Rejection after Heart Transplantation. PLoS One. 2017;12(1):e0170842. Published 2017 Jan 26.
48. Zhao Z, Qu F, Liu R, Xia Y. Differential expression of miR-142-3p protects cardiomyocytes from myocardial ischemia-reperfusion via TLR4/NFkB axis [published online ahead of print, 2019 Nov 20]. J Cell Biochem. 2019;10.1002/jcb.29506.
49. Cao ML, Zhu BL, Sun YY, Qiu GR, Fu WN, Jiang HK. MicroRNA-144 Regulates Cardiomyocyte Proliferation and Apoptosis by Targeting TBX1 through the JAK2/STAT1 Pathway. Cytogenet Genome Res. 2019;159(4):190-200.
50. Gan M, Zhang S, Fan Y, et al. The Expression of microRNA in Adult Rat Heart with Isoproterenol-Induced Cardiac Hypertrophy. Cells. 2020;9(5):1173. Published 2020 May 8.
51. Yuan X, Pan J, Wen L, et al. MiR-144-3p Enhances Cardiac Fibrosis After Myocardial Infarction by Targeting PTEN. Front Cell Dev Biol. 2019;7:249. Published 2019 Oct 29.
52. Yang P, Yang Y, He X, et al. miR-153-3p Targets βII Spectrin to Regulate Formaldehyde-Induced Cardiomyocyte Apoptosis. Front Cardiovasc Med. 2021;8:764831. Published 2021 Dec 15.
53. Chen A, Wen J, Lu C, et al. Inhibition of miR 155 5p attenuates the valvular damage induced by rheumatic heart disease. Int J Mol Med. 2020;45(2):429-440.
54. Yang Y, Zhou Y, Cao Z, et al. miR-155 functions downstream of angiotensin II receptor subtype 1 and calcineurin to regulate cardiac hypertrophy. Exp Ther Med. 2016;12(3):1556-1562.
55. Hu J, Huang CX, Rao PP, et al. MicroRNA-155 inhibition attenuates endoplasmic reticulum stress-induced cardiomyocyte apoptosis following myocardial infarction via reducing macrophage inflammation. Eur J Pharmacol. 2019;857:172449.
56. Copier CU, León L, Fernández M, Contador D, Calligaris SD. Circulating miR-19b and miR-181b are potential biomarkers for diabetic cardiomyopathy. Sci Rep. 2017;7(1):13514. Published 2017 Oct 18.
57. Yuan L, Fan L, Li Q, Cui W, Wang X, Zhang Z. Inhibition of miR-181b-5p protects cardiomyocytes against ischemia/reperfusion injury by targeting AKT3 and PI3KR3. J Cell Biochem. 2019;120(12):19647-19659.
58. Zhang Y, Peng B, Han Y. MiR-182 alleviates the development of cyanotic congenital heart disease by suppressing HES1. Eur J Pharmacol. 2018;836:18-24.
59. Zhang Y, Fang J, Ma H. Inhibition of miR-182-5p protects cardiomyocytes from hypoxia-induced apoptosis by targeting CIAPIN1. Biochem Cell Biol. 2018;96(5):646-654.
60. Tao Y, Zhang H, Huang S, et al. miR-199a-3p promotes cardiomyocyte proliferation by inhibiting Cd151 expression. Biochem Biophys Res Commun. 2019;516(1):28-36.
61. Li Z, Song Y, Liu L, et al. miR-199a impairs autophagy and induces cardiac hypertrophy through mTOR activation. Cell Death Differ. 2017;24(7):1205-1213.
62. Asensio-Lopez MC, Sassi Y, Soler F, Fernandez Del Palacio MJ, Pascual-Figal D, Lax A. The miRNA199a/SIRT1/P300/Yy1/sST2 signaling axis regulates adverse cardiac remodeling following MI. Sci Rep. 2021;11(1):3915. Published 2021 Feb 16.
63. Obradovic D, Rommel KP, Blazek S, et al. The potential role of plasma miR-155 and miR-206 as circulatory biomarkers in inflammatory cardiomyopathy. ESC Heart Fail. 2021;8(3):1850-1860.
64. Yang Y, Del Re DP, Nakano N, et al. miR-206 Mediates YAP-Induced Cardiac Hypertrophy and Survival. Circ Res. 2015;117(10):891-904.
65. Yan Y, Dang H, Zhang X, Wang X, Liu X. The protective role of MiR-206 in regulating cardiomyocytes apoptosis induced by ischemic injury by targeting PTP1B. Biosci Rep. 2020;40(1):BSR20191000.
66. Zhou Q, Schötterl S, Backes D, et al. Inhibition of miR-208b improves cardiac function in titin-based dilated cardiomyopathy. Int J Cardiol. 2017;230:634-641.
67. Soci UPR, Fernandes T, Barauna VG, et al. Epigenetic control of exercise training-induced cardiac hypertrophy by miR-208. Clin Sci (Lond). 2016;130(22):2005-2015.
68. Liu X, Yuan L, Chen F, et al. Circulating miR-208b: A Potentially Sensitive and Reliable Biomarker for the Diagnosis and Prognosis of Acute Myocardial Infarction. Clin Lab. 2017;63(1):101-109.
69. Yin Y, Lv L, Wang W. Expression of miRNA-214 in the sera of elderly patients with acute myocardial infarction and its effect on cardiomyocyte apoptosis. Exp Ther Med. 2019;17(6):4657-4662.
70. Yang T, Gu H, Chen X, et al. Cardiac hypertrophy and dysfunction induced by overexpression of miR-214 in vivo. J Surg Res. 2014;192(2):317-325.
71. Yang K, Shi J, Hu Z, Hu X. The deficiency of miR-214-3p exacerbates cardiac fibrosis via miR-214-3p/NLRC5 axis. Clin Sci (Lond). 2019;133(17):1845-1856. Published 2019 Sep 10.
72. Li F, Li SS, Chen H, et al. miR-320 accelerates chronic heart failure with cardiac fibrosis through activation of the IL6/STAT3 axis. Aging (Albany NY). 2021;13(18):22516-22527.
73. Tian ZQ, Jiang H, Lu ZB. MiR-320 regulates cardiomyocyte apoptosis induced by ischemia-reperfusion injury by targeting AKIP1. Cell Mol Biol Lett. 2018;23:41. Published 2018 Aug 28.
74. Li K, Lin Y, Li C. MiR-338-5p ameliorates pathological cardiac hypertrophy by targeting CAMKIIδ. Arch Pharm Res. 2019;42(12):1071-1080.
75. Fu DL, Jiang H, Li CY, Gao T, Liu MR, Li HW. MicroRNA-338 in MSCs-derived exosomes inhibits cardiomyocyte apoptosis in myocardial infarction. Eur Rev Med Pharmacol Sci. 2020;24(19):10107-10117.
76. Zhou J, Gao J, Zhang X, et al. microRNA-340-5p Functions Downstream of Cardiotrophin-1 to Regulate Cardiac Eccentric Hypertrophy and Heart Failure via Target Gene Dystrophin. Int Heart J. 2015;56(4):454-458.
77. Li D, Zhou J, Yang B, Yu Y. microRNA-340-5p inhibits hypoxia/reoxygenation-induced apoptosis and oxidative stress in cardiomyocytes by regulating the Act1/NF-κB pathway. J Cell Biochem. 2019;120(9):14618-14627.
78. Shen X, Soibam B, Benham A, et al. miR-322/-503 cluster is expressed in the earliest cardiac progenitor cells and drives cardiomyocyte specification. Proc Natl Acad Sci U S A. 2016;113(34):9551-9556.
79. Marchand A, Atassi F, Mougenot N, et al. miR-322 regulates insulin signaling pathway and protects against metabolic syndrome-induced cardiac dysfunction in mice. Biochim Biophys Acta. 2016;1862(4):611-621.
80. Baptista R, Marques C, Catarino S, et al. MicroRNA-424(322) as a new marker of disease progression in pulmonary arterial hypertension and its role in right ventricular hypertrophy by targeting SMURF1. Cardiovasc Res.
81. Chen Z, Su X, Shen Y, et al. MiR322 mediates cardioprotection against ischemia/reperfusion injury via FBXW7/notch pathway. J Mol Cell Cardiol. 2019;133:67-74.
82. Lange S, Banerjee I, Carrion K, et al. miR-486 is modulated by stretch and increases ventricular growth. JCI Insight. 2019;4(19):e125507. Published 2019 Sep 12.
83. Zhang R, Lan C, Pei H, Duan G, Huang L, Li L. Expression of circulating miR-486 and miR-150 in patients with acute myocardial infarction. BMC Cardiovasc Disord. 2015;15:51. Published 2015 Jun 16.
84. Wang X, Zhang X, Ren XP, et al. MicroRNA-494 targeting both proapoptotic and antiapoptotic proteins protects against ischemia/reperfusion-induced cardiac injury. Circulation. 2010;122(13):1308-1318.
85. Jafarzadeh M, Mohammad Soltani B, Ekhteraei Tousi S, Behmanesh M. Hsa-miR-497 as a new regulator in TGFβ signaling pathway and cardiac differentiation process. Gene. 2018;675:150-156.
86. Xiao Y, Zhang X, Fan S, Cui G, Shen Z. MicroRNA-497 Inhibits Cardiac Hypertrophy by Targeting Sirt4. PLoS One. 2016;11(12):e0168078. Published 2016 Dec 16.
87. Jin K, Wang B, Ruan ZB, Chen GC, Ren Y. Effect of miR-497 on myocardial cell apoptosis in rats with myocardial ischemia/reperfusion through the MAPK/ERK signaling pathway. Eur Rev Med Pharmacol Sci. 2019;23(19):8580-8587.
