# Supplementary material for: Left ventricle function and post-transcriptional events with exercise training in pigs
Source: PLoS One. 2024 Feb 2;19(2):e0292243. doi: 10.1371/journal.pone.0292243 (PMC10836705; doi:10.1371/journal.pone.0292243)
Supplement: S2 Table — (DOCX) [file pone.0292243.s002.docx]

| **Supplemental Table 2: Association Between Myocardial miR Levels & Stiffness Indices for all miRs with a Greater Than 2 Fold Change** | | | |
| --- | --- | --- | --- |
|  | | **Regional Stiffness** | **LV Stiffness** |
| **ssc-miR-1** | Pearson Correlation | -0.098 | -0.357 |
|  | Sig. (2-tailed) | 0.728 | 0.191 |
| **ssc-let-7a** | Pearson Correlation | -0.303 | -0.468 |
|  | Sig. (2-tailed) | 0.273 | 0.078 |
| **ssc-let-7c** | Pearson Correlation | -0.422 | -0.492 |
|  | Sig. (2-tailed) | 0.118 | 0.062 |
| **ssc-let-7d-5p** | Pearson Correlation | -0.282 | -0.496 |
|  | Sig. (2-tailed) | 0.309 | 0.060 |
| **ssc-let-7e** | Pearson Correlation | -0.218 | -0.462 |
|  | Sig. (2-tailed) | 0.434 | 0.083 |
| **ssc-let-7f** | Pearson Correlation | -0.120 | -0.398 |
|  | Sig. (2-tailed) | 0.670 | 0.142 |
| **ssc-miR-15b** | Pearson Correlation | -0.282 | -0.508 |
|  | Sig. (2-tailed) | 0.308 | 0.053 |
| **ssc-miR-19a** | Pearson Correlation | 0.263 | 0.543 |
|  | Sig. (2-tailed) | 0.344 | 0.037 |
| **ssc-miR-22-3p** | Pearson Correlation | 0.283 | 0.646 |
|  | Sig. (2-tailed) | 0.307 | 0.009 |
| **ssc-miR-23a** | Pearson Correlation | -0.124 | -0.322 |
|  | Sig. (2-tailed) | 0.659 | 0.242 |
| **ssc-miR-23b** | Pearson Correlation | -0.124 | -0.396 |
|  | Sig. (2-tailed) | 0.659 | 0.144 |
| **ssc-miR-30e-5p** | Pearson Correlation | 0.579 | 0.671 |
|  | Sig. (2-tailed) | 0.024 | 0.006 |
| **ssc-miR-31** | Pearson Correlation | 0.182 | -0.072 |
|  | Sig. (2-tailed) | 0.534 | 0.806 |
| **ssc-miR-98** | Pearson Correlation | -0.152 | -0.422 |
|  | Sig. (2-tailed) | 0.589 | 0.117 |
| **ssc-miR-99a** | Pearson Correlation | 0.515 | 0.638 |
|  | Sig. (2-tailed) | 0.050 | 0.010 |
| **ssc-miR-124a** | Pearson Correlation | 0.195 | -0.146 |
|  | Sig. (2-tailed) | 0.565 | 0.668 |
| **ssc-miR-133a** | Pearson Correlation | 0.099 | 0.173 |
|  | Sig. (2-tailed) | 0.727 | 0.538 |
| **ssc-miR-142** | Pearson Correlation | 0.388 | 0.814 |
|  | Sig. (2-tailed) | 0.153 | 0.000 |
| **ssc-miR-144** | Pearson Correlation | 0.256 | 0.613 |
|  | Sig. (2-tailed) | 0.399 | 0.026 |
| **ssc-miR-155-5p** | Pearson Correlation | -0.119 | -0.358 |
|  | Sig. (2-tailed) | 0.673 | 0.190 |
| **ssc-miR-181b** | Pearson Correlation | -0.303 | -0.429 |
|  | Sig. (2-tailed) | 0.272 | 0.111 |
| **ssc-miR-182** | Pearson Correlation | 0.185 | -0.147 |
|  | Sig. (2-tailed) | 0.509 | 0.601 |
| **ssc-miR-199a-5p** | Pearson Correlation | 0.078 | 0.575 |
|  | Sig. (2-tailed) | 0.782 | 0.025 |
| **ssc-miR-206** | Pearson Correlation | -0.026 | -0.281 |
|  | Sig. (2-tailed) | 0.926 | 0.310 |
| **ssc-miR-208b** | Pearson Correlation | 0.156 | 0.452 |
|  | Sig. (2-tailed) | 0.579 | 0.091 |
| **ssc-miR-214** | Pearson Correlation | -0.244 | -0.351 |
|  | Sig. (2-tailed) | 0.381 | 0.199 |
| **ssc-miR-320** | Pearson Correlation | -0.445 | -0.441 |
|  | Sig. (2-tailed) | 0.096 | 0.100 |
| **ssc-miR-338** | Pearson Correlation | 0.046 | 0.359 |
|  | Sig. (2-tailed) | 0.871 | 0.189 |
| **ssc-miR-340** | Pearson Correlation | 0.348 | 0.412 |
|  | Sig. (2-tailed) | 0.203 | 0.128 |
| **ssc-miR-424** | Pearson Correlation | 0.396 | 0.288 |
|  | Sig. (2-tailed) | 0.143 | 0.297 |
| **ssc-miR-486** | Pearson Correlation | -0.287 | -0.496 |
|  | Sig. (2-tailed) | 0.300 | 0.060 |
| **ssc-miR-497** | Pearson Correlation | 0.391 | 0.726 |
|  | Sig. (2-tailed) | 0.149 | 0.002 |
