# Supplementary material for: Left ventricle function and post-transcriptional events with exercise training in pigs
Source: PLoS One. 2024 Feb 2;19(2):e0292243. doi: 10.1371/journal.pone.0292243 (PMC10836705; doi:10.1371/journal.pone.0292243)
Supplement: S3 Table — (DOCX) [file pone.0292243.s003.docx]

**Table 3b. miRs with Association to LV Stiffness Indices and Functional Domain**

| **miR** | **Inflammation** | **ECM** | **Calcium Handling** |
| --- | --- | --- | --- |
| **ssc-miR-19a** | 1 | 2 | 3 |
| **ssc-miR-22** | 4 | 5 | 6 |
| **ssc-miR-30e** | 7 | 8 |  |
| **ssc-miR-99a** | 9 |  |  |
| **ssc-miR-142** | 10 | 11 | 12 |
| **ssc-miR-144** | 13 | 14 | 15 |
| **ssc-miR-199a** | 16 | 17 | 18 |
| **ssc-miR-497** |  | 19 |  |

1. Chen, H., Li, X., Liu, S., Gu, L., & Zhou, X. (2017). MircroRNA-19a promotes vascular inflammation and foam cell formation by targeting HBP-1 in atherogenesis. Scientific reports, 7(1), 12089. <https://doi.org/10.1038/s41598-017-12167-z>
2. Zou, M., Wang, F., Gao, R., Wu, J., Ou, Y., Chen, X., Wang, T., Zhou, X., Zhu, W., Li, P., Qi, L. W., Jiang, T., Wang, W., Li, C., Chen, J., He, Q., & Chen, Y. (2016). Autophagy inhibition of hsa-miR-19a-3p/19b-3p by targeting TGF-β R II during TGF-β1-induced fibrogenesis in human cardiac fibroblasts. Scientific reports, 6, 24747. <https://doi.org/10.1038/srep24747>
3. Ma, J., Chen, Z., Ma, Y., Xia, Y., Hu, K., Zhou, Y., Chen, A., Qian, J., & Ge, J. (2020). MicroRNA-19a attenuates hypoxia-induced cardiomyocyte apoptosis by downregulating NHE-1 expression and decreasing calcium overload. Journal of cellular biochemistry, 121(2), 1747–1758. <https://doi.org/10.1002/jcb.29411>
4. Gu, W., Zhan, H., Zhou, X. Y., Yao, L., Yan, M., Chen, A., Liu, J., Ren, X., Zhang, X., Liu, J. X., & Liu, G. (2017). MicroRNA-22 regulates inflammation and angiogenesis via targeting VE-cadherin. FEBS letters, 591(3), 513–526. <https://doi.org/10.1002/1873-3468.12565>
5. Hong, Y., Cao, H., Wang, Q., Ye, J., Sui, L., Feng, J., Cai, X., Song, H., Zhang, X., & Chen, X. (2016). MiR-22 may Suppress Fibrogenesis by Targeting TGFβR I in Cardiac Fibroblasts. Cellular physiology and biochemistry : international journal of experimental cellular physiology, biochemistry, and pharmacology, 40(6), 1345–1353. <https://doi.org/10.1159/000453187>
6. Gurha, P., Abreu-Goodger, C., Wang, T., Ramirez, M. O., Drumond, A. L., van Dongen, S., Chen, Y., Bartonicek, N., Enright, A. J., Lee, B., Kelm, R. J., Jr, Reddy, A. K., Taffet, G. E., Bradley, A., Wehrens, X. H., Entman, M. L., & Rodriguez, A. (2012). Targeted deletion of microRNA-22 promotes stress-induced cardiac dilation and contractile dysfunction. Circulation, 125(22), 2751–2761. <https://doi.org/10.1161/CIRCULATIONAHA.111.044354>
7. Dai, R., Ren, Y., Lv, X., Chang, C., He, S., Li, Q., Yang, X., Ren, L., Wei, R., & Su, Q. (2023). MicroRNA-30e-3p reduces coronary microembolism-induced cardiomyocyte pyroptosis and inflammation by sequestering HDAC2 from the SMAD7 promoter. American journal of physiology. Cell physiology, 324(2), C222–C235. <https://doi.org/10.1152/ajpcell.00351.2022>
8. Zhang, W., Chang, H., Zhang, H., & Zhang, L. (2017). MiR-30e Attenuates Isoproterenol-induced Cardiac Fibrosis Through Suppressing Snai1/TGF-β Signaling. Journal of cardiovascular pharmacology, 70(6), 362–368. <https://doi.org/10.1097/FJC.0000000000000526>
9. Wang, G., Jing, S. Y., Liu, G., Guo, X. J., Zhao, W., Jia, X. L., Zheng, M. Q., & Tan, W. Y. (2022). miR-99a-5p: A Potential New Therapy for Atherosclerosis by Targeting mTOR and Then Inhibiting NLRP3 Inflammasome Activation and Promoting Macrophage Autophagy. Disease markers, 2022, 7172583. <https://doi.org/10.1155/2022/7172583>
10. Sharma, S., Liu, J., Wei, J., Yuan, H., Zhang, T., & Bishopric, N. H. (2012). Repression of miR-142 by p300 and MAPK is required for survival signalling via gp130 during adaptive hypertrophy. EMBO molecular medicine, 4(7), 617–632. <https://doi.org/10.1002/emmm.201200234>
11. Wang, Z., Fu, M., & Li, Y. (2020). miR-142-5p and miR-212-5p cooperatively inhibit the proliferation and collagen formation of cardiac fibroblasts by regulating c-Myc/TP53INP1. Canadian journal of physiology and pharmacology, 98(5), 314–323. <https://doi.org/10.1139/cjpp-2019-0495>
12. Nafzger, S., & Rougier, J. S. (2017). Calcium/calmodulin-dependent serine protein kinase CASK modulates the L-type calcium current. Cell calcium, 61, 10–21.
13. Yang, G., Tang, X., Tan, L., Nong, D., Yang, P., & Ning, H. (2021). Upregulation of miR-144-3p protects myocardial function from ischemia-reperfusion injury through inhibition of TMEM16A Ca2+-activated chloride channel. Human cell, 34(2), 360–371. <https://doi.org/10.1007/s13577-020-00482-z>
14. Yuan, X., Pan, J., Wen, L., Gong, B., Li, J., Gao, H., Tan, W., Liang, S., Zhang, H., & Wang, X. (2019). MiR-144-3p Enhances Cardiac Fibrosis After Myocardial Infarction by Targeting PTEN. Frontiers in cell and developmental biology, 7, 249. <https://doi.org/10.3389/fcell.2019.00249>
15. Turczyńska, K. M., Bhattachariya, A., Säll, J., Göransson, O., Swärd, K., Hellstrand, P., & Albinsson, S. (2013). Stretch-sensitive down-regulation of the miR-144/451 cluster in vascular smooth muscle and its role in AMP-activated protein kinase signaling. PloS one, 8(5), e65135. <https://doi.org/10.1371/journal.pone.0065135>
16. Liu, M., Cao, Y., Hu, Y., Zhang, Z., Ji, S., Shi, L., & Tao, G. (2022). MiR-199a-3p Restrains Foaming and Inflammation by Regulating RUNX1 in Macrophages. Molecular biotechnology, 64(10), 1130–1142. <https://doi.org/10.1007/s12033-022-00484-2>
17. Zeng, N., Huang, Y. Q., Yan, Y. M., Hu, Z. Q., Zhang, Z., Feng, J. X., Guo, J. S., Zhu, J. N., Fu, Y. H., Wang, X. P., Zhang, M. Z., Duan, J. Z., Zheng, X. L., Xu, J. D., & Shan, Z. X. (2021). Diverging targets mediate the pathological roleof miR-199a-5p and miR-199a-3p by promoting cardiac hypertrophy and fibrosis. Molecular therapy. Nucleic acids, 26, 1035–1050. <https://doi.org/10.1016/j.omtn.2021.10.013>
18. Garcia-Elias, A., Tajes, M., Yañez-Bisbe, L., Enjuanes, C., Comín-Colet, J., Serra, S. A., Fernández-Fernández, J. M., Aguilar-Agon, K. W., Reilly, S., Martí-Almor, J., & Benito, B. (2021). Atrial Fibrillation in Heart Failure Is Associated with High Levels of Circulating microRNA-199a-5p and 22-5p and a Defective Regulation of Intracellular Calcium and Cell-to-Cell Communication. International journal of molecular sciences, 22(19), 10377. <https://doi.org/10.3390/ijms221910377>
19. Xiao, Y., Zhang, X., Fan, S., Cui, G., & Shen, Z. (2016). MicroRNA-497 Inhibits Cardiac Hypertrophy by Targeting Sirt4. PloS one, 11(12), e0168078. https://doi.org/10.1371/journal.pone.0168078
