# Supplementary figures and images for: Left ventricle function and post-transcriptional events with exercise training in pigs
Source: PLoS One. 2024 Feb 2;19(2):e0292243. doi: 10.1371/journal.pone.0292243 (PMC10836705; doi:10.1371/journal.pone.0292243)

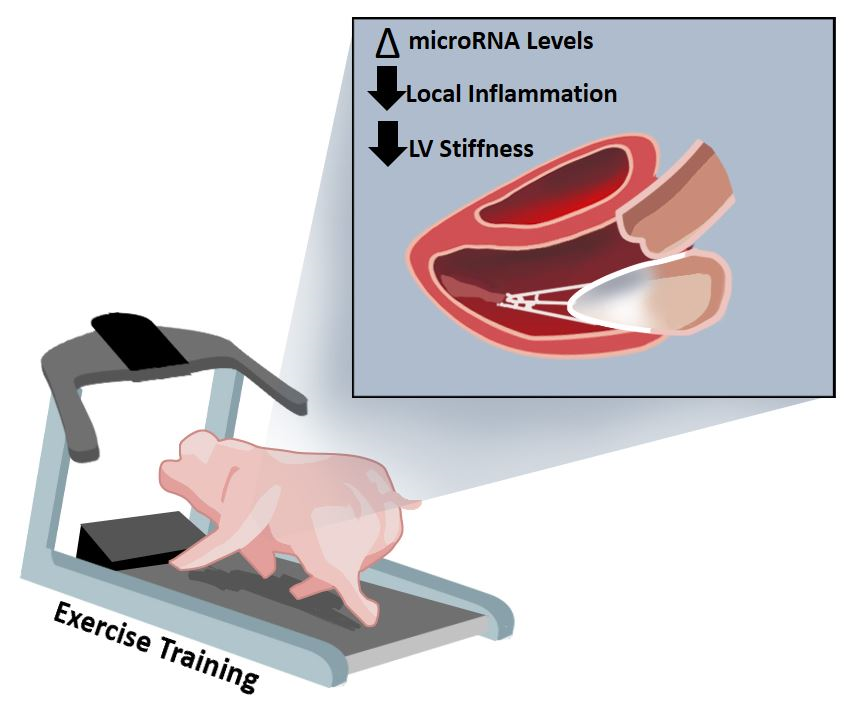

Supplement: S1 Graphical abstract — (TIF) [file pone.0292243.s004.tif]
